# In-hospital medical complications associated with stroke recurrence after initial ischemic stroke: A prospective cohort study from the China National Stroke Registry

**DOI:** 10.1097/MD.0000000000004929

**Published:** 2016-09-16

**Authors:** Penglian Wang, Yilong Wang, Xingquan Zhao, Wanliang Du, Anxin Wang, Gaifen Liu, Liping Liu, Ruijun Ji, Chunxue Wang, Kehui Dong, Yongjun Wang

**Affiliations:** a Department of Neurology, Beijing Tiantan Hospital, Capital Medical University; b Center of Stroke, Beijing Institute for Brain Disorders; c Beijing Key Laboratory of Translational Medicine for Cerebrovascular Disease; d China National Clinical Research Center for Neurological Diseases, Beijing, China.

**Keywords:** complication, ischemic stroke, outcome, recurrent stroke

## Abstract

In-hospital medical complications are common and strongly associated with the risk of death and dependency in stroke patients. Whether similar associations extend to stroke recurrence is unclear. We prospectively and systematically investigated whether in-hospital medical complications are associated with recurrent stroke of patients in the China National Stroke Registry (CNSR). We examined patients with initial ischemic stroke enrolled in CNSR between 2007 and 2008. Recurrent stroke at 3, 6, and 12 months post-stroke was used as stroke outcome. Medical complications were associated with stroke outcomes using multivariable logistic regression.

Of the 7593 study patients, recurrent stroke occurred in 1115 (14.7%) within 12 months after stroke onset. In-hospital medical complications were independent risk factors for stroke recurrence in patients with initial ischemic stroke at 3 months (adjusted odds ratio (OR) = 2.19, 95% confidence interval (CI) 1.85 to 2.60), 6 months (adjusted OR = 2.04, 95% CI 1.74 to 2.38), and 12 months (adjusted OR = 1.88; 95% CI 1.62 to 2.19) after onset. The persistence of secondary prevention medications in patients with complications was lower than that in patients without complications.

Stroke recurrence post-acute ischemic stroke is significantly associated with in-hospital medical complications.

## Abstract 2

Supplemental Digital Content is available in the text

## 1 Introduction

Stroke is a major cause of disability and death worldwide,^[1–5]^ affecting 7 million people each year in China.^[2]^ Although initial stroke events play a principal role in the outcome of stroke, recurrent stroke events are relevant to higher mortality rates and disability levels.^[6]^ Given these results, identifying factors that are associated with recurrent stroke is critical. Stroke recurrence is also an important factor in evaluating the quality of health care services. Several studies have identified potential predictors of recurrent stroke, including age,^[7–12]^ race,^[11]^ hypertension,^[8,13–17]^ diabetes mellitus,^[7,9,13,17,18]^ atrial fibrillation,^[8,10,14,16]^ ethanol abuse,^[17,19]^ smoking,^[16]^ hyperlipidemia,^[15]^ elevated homocysteine,^[20]^ patent foramen ovale,^[21]^ and metabolic syndrome.^[22]^ In-hospital medical complications represent potentially modifiable factors contributing to recurrent stroke events. Previous studies have demonstrated that medical complications are strong risk factors for poor outcome in patients with stroke.^[23–31]^ Data from the China National Stroke Registry (CNSR) further confirmed that in-hospital medical complications were independently associated with a greater risk of death or dependency in patients with stroke.^[32,33]^ Despite this evidence, little is known about the association of medical complications with recurrent stroke. We therefore hypothesized that the presence of medical complications during hospitalization predicts stroke recurrence within 12 months after initial ischemic stroke.

## 2 Methods

### 2.1 Data collection and study population

The CNSR study is a national, prospective cohort investigation of patient characteristics, risk factors, the stroke care system, and the outcomes of patients with acute stroke in China. Details of the study design have been reviewed previously.^[34]^ Patients with acute stroke were eligible for this prospective registry study if they met the following recruitment criteria: adults of either sex, acute stroke diagnosed using World Health Organization ICD-10 criteria and brain CT or MRI confirmation, patients within the first 14 days after onset of stroke, and written informed consent obtained from patients or surrogate. Patients with initial ischemic stroke were included in this analysis. Enrolled patients who were diagnosed with recurrent ischemic stroke, intracerebral hemorrhage, subarachnoid hemorrhage, or transient ischemic attack (TIA) and refused follow-up were excluded in this analysis. The recruitment period for the CNSR study was between September 2007 and August 2008, and follow-ups were completed in August 2009. We collected data at baseline, at discharge, and at 3, 6, and 12 months after stroke onset. At baseline, every patient was interviewed and evaluated by a trained investigator who recorded demographic information, medical history, family history of stroke, baseline National Institutes of Health Stroke Scale (NIHSS) scores, and other clinical characteristics. The baseline severity of neurological impairment was determined by baseline NIHSS score.^[35]^ During hospitalization, complications, treatments, and other relative information were recorded. Details of secondary prevention medications and other clinical characteristics were recorded at discharge and at 3, 6, and 12 months after patients were recruited. The information was usually obtained from the patient, otherwise the details of the patient were obtained from surrogate or caregiver. Patients were divided into two groups: those with and without in-hospital medical complications.

### 2.2 Definition of medical complications

All in-hospital medical complications were clarified as non-neurological complications that occurred during the in-hospital period and required an intervention. Pneumonia, urinary tract infection, gastrointestinal bleeding, decubitus ulcer, deep vein thrombosis, and pulmonary embolism were included according to paper-based registry forms. The same criteria were used for diagnosing in-hospital medical complications by all the participating clinical centers to record each medical complication. The in-hospital medical complications were defined as described previously (Supplementary Table 1).^[32]^

### 2.3 Ascertainment of outcomes

Recurrent stroke was used as clinical outcome in this analysis. The outcomes of interest were defined as having either a new neurological deficit or a new deterioration of a previous deficit, including ischemic, hemorrhagic, or undetermined stroke (according to WHO criteria) from hospital discharge to 12-month follow-up assessment. Certificates were faxed to Beijing Tiantan Hospital when stroke recurrence was reported by the patient, surrogate, or caregiver during follow-up. A judgement was required for any suspected stroke recurrence by the research coordinators as well as the principal investigator.

### 2.4 Definition of medication(s) persistence

Persistence to secondary prevention medications was determined for the 3 types of medications: antiplatelet agents (including aspirin or clopidogrel), antihypertensive agents (including calcium-channel blockers, angiotensin converting enzyme inhibitors, angiotensin receptor blockers, beta-blockers, or diuretics), and diabetic agents (insulin or oral agents). Standard scripts were used to collect the data on medication(s) persistence. Medication(s) information at discharge after disease onset was considered as the reference value for assessing persistence. Persistence to medications was defined as medication(s) continuation during discharge and 12-month post-onset.

### 2.5 Statistical analysis

Categorical and continuous variables were respectively expressed as number with proportions, and mean with standard deviation (SD) or median with interquartile range (IQR). Differences between patients with complications and patients without complications were tested by using a *t*-test for continuous variables and the χ^2^ test for categorical variables. For identifying associations between the patient characteristics and stroke recurrence, univariate logistic regression analyses were performed and presented as unadjusted odds ratios (ORs) with their corresponding 95% confidence intervals (CIs). For identifying associations between in-hospital medical complications and stroke recurrence, univariate logistic regression analyses and multivariate logistic regression analyses were performed and presented as unadjusted ORs with their corresponding 95% CIs and adjusted ORs with the 95% CIs. Adjusted potential covariates included age, sex, baseline NIHSS, hypertension, diabetes mellitus, hyperlipidemia, history of coronary heart disease, family history of stroke, atrial fibrillation, current smoking status, dysphagia, heavy alcohol intake, anticoagulant treatment, thrombolytic treatment, and types of health insurance. Missing values are treated as the most categories or the categories based on clinical perspective. SAS statistical software, version 9.4 (SAS Institute Inc., Cary, NC) was used to analyze data, considering statistical significance by a two-tailed probability value of < 0.05.

### 2.6 Standard protocol approvals and patient consents

The study design and procedures were approved by the institutional review in each participating hospital before screening subjects for CNSR. Written informed consent was obtained from all patients or surrogates of patients recruited in the study.

## 3 Results

**Figure 1:**
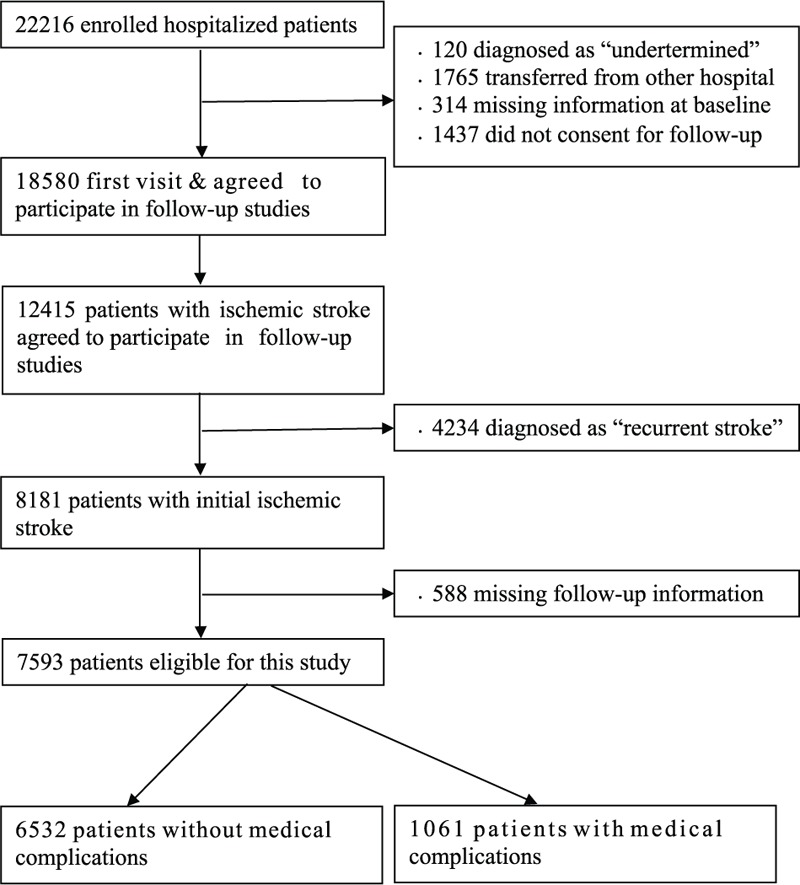
Flow diagram of study population.

Overall, 22,216 hospitalized patients with stroke from 132 sites were enrolled in the CNSR study. Of these, 18,580 stroke patients agreed to participate in the follow-up studies following their first visit, of which there were 12,415 patients with ischemic stroke. We excluded 4234 individuals with recurrent stroke and 588 individuals missing follow-up data, leaving 7593 participants with initial ischemic stroke in the final analysis (Fig. 1). The prevalence of pneumonia, urinary tract infection, gastrointestinal bleeding, decubitus ulcer, deep vein thrombosis, and pulmonary embolism during hospital was 10.6%, 3.2%, 2.2%, 0.5%, 0.4%, and 0.3%, respectively. Of 7593 patients, 1061 (13.9%) patients had an in-hospital medical complication(s).

We reported the baseline characteristics of participants having initial ischemic stroke without or with in-hospital medical complications (Table 1). The median age was 64, and 61.4% were male. In general, compared with participants without in-hospital medical complications, participants with complications were significantly more likely to be older; to have a history of coronary heart disease, atrial fibrillation, and higher NIHSS scores; and to be receiving anticoagulants or thrombolytic treatment. Participants with complications were less likely to have diabetes mellitus and a family history of stroke. There were no significant differences in the proportion of hypertension, hyperlipidemia, history of TIA, and types of health insurance for participants with or without complications.

**Table 1:**
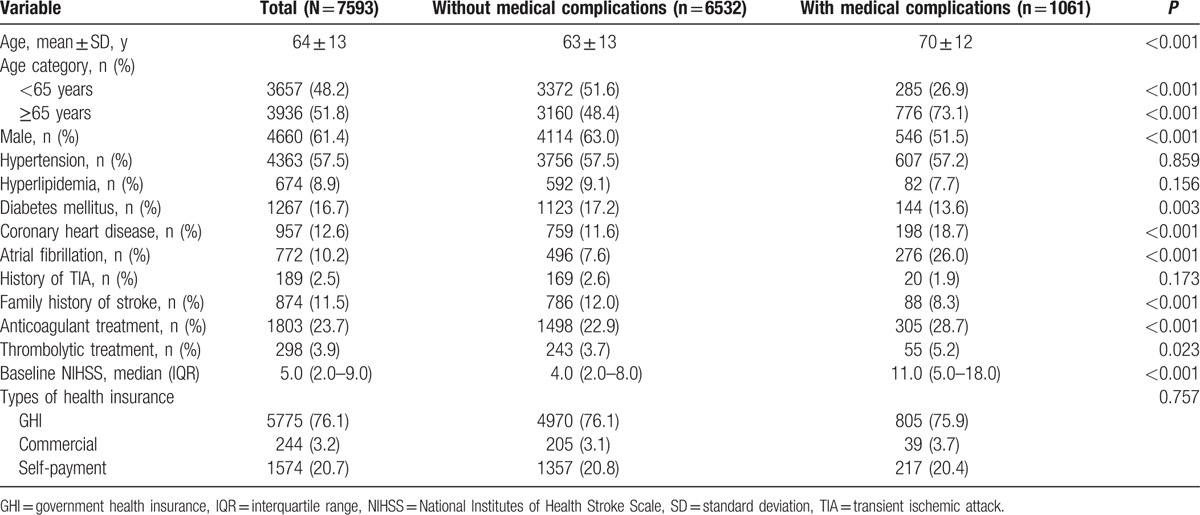
Baseline characteristics of patients with initial ischemic stroke (N = 7593).

**Figure 2:**
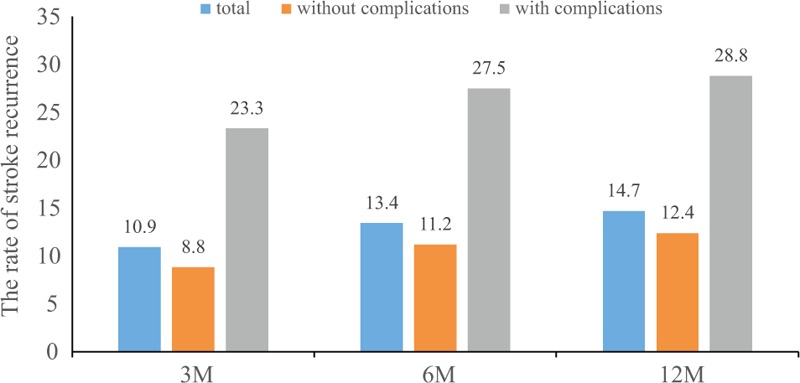
The clinical outcomes of the study at 3, 6, and 12 months after disease onset. M = months.

The clinical outcomes of this study at 3, 6, and 12 months after stroke are shown in Fig. 2. The rate of recurrent stroke at 3, 6, and 12 months after initial ischemic stroke was 10.9% (824 patients), 13.4% (1019 patients), and 14.7% (1115 patients), respectively. The rate of recurrent stroke was significantly higher in participants with complications than that in patients without complications at all-time points (Fig. 2).

**Figure 3:**
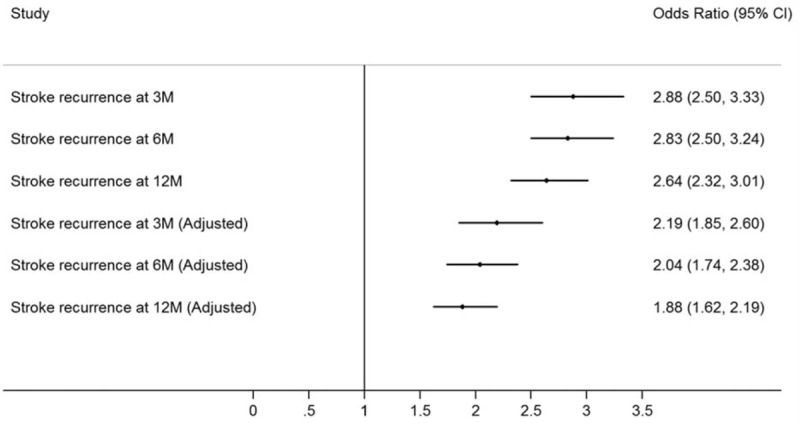
Unadjusted and adjusted odds ratios of presenting any in-hospital medical complications for stroke recurrence following initial ischemic stroke. CI = confidence interval, M = months, NIHSS = National Institutes of Health Stroke Scale, TIA = transient ischemic attack. ^∗^Adjusted for age, sex, baseline NIHSS, hypertension, diabetes mellitus, hyperlipidemia, history of coronary heart disease, history of stroke, history of TIA, family history of stroke, atrial fibrillation, current smoking status, dysphagia, heavy alcohol intake, anticoagulant treatment, thrombolytic treatment, and types of health insurance.

The univariate logistic regression analysis showed that age, diabetes mellitus, coronary heart disease, atrial fibrillation, history of TIA, having anticoagulants, having thrombolytic treatment, baseline NIHSS, and types of health insurance were significantly associated with recurrent stroke at 3, 6, or/and 12 months after stroke (Table 2). In-hospital medical complications were associated with greater risk of recurrent stroke events in analyses adjusted for age, sex, baseline NIHSS, hypertension, diabetes mellitus, hyperlipidemia, history of coronary heart disease, history of TIA, family history of stroke, atrial fibrillation, current smoking status, dysphagia, heavy alcohol intake, anticoagulant treatment, thrombolytic treatment, and types of health insurance (Fig. 3). The adjusted ORs of recurrent stroke were approximately 2.2-fold, 2.0-fold, and 1.9-fold greater in participants with complications when compared with those without complications at 3, 6, and 12 months, respectively.

**Table 2:**
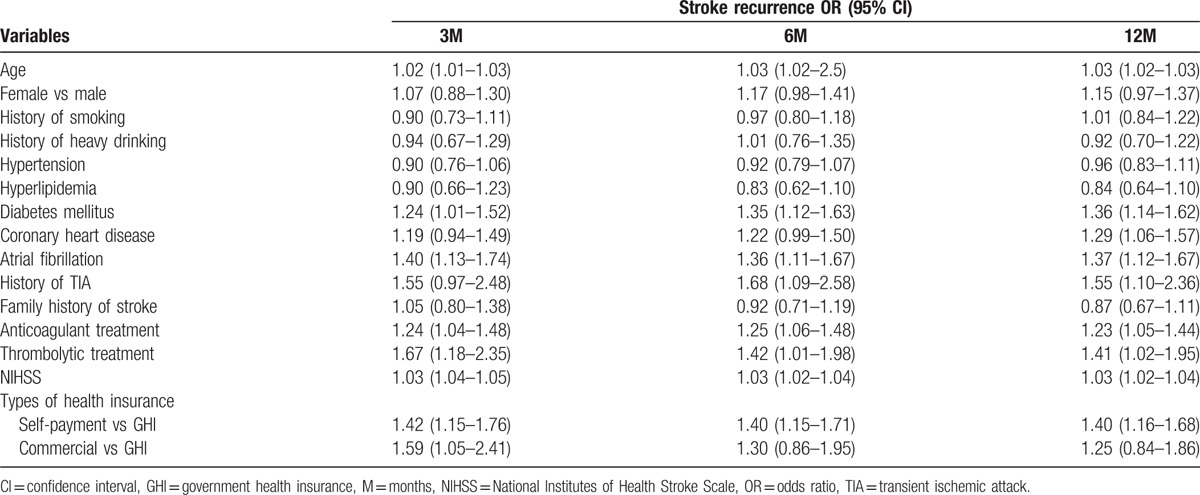
Univariate logistic regression analyzing the effect of confounders on stroke recurrence at 3, 6, and 12 months after initial ischemic stroke.

To evaluate possible explanations for the effect of in-hospital medical complication on stroke recurrence after acute ischemic stroke, we compared differences of patients with or without in-hospital medical complications in the persistence for secondary prevention associated with stroke recurrence (Table 3). By medication class, 12-month persistence was highest for antihypertensive agents (72.7%), followed by diabetic (66.2%), and antiplatelet (54.9%) agents (Table 3). Patients with in-hospital medical complications were less likely to have persistence of antihypertensive, diabetic, and antiplatelet agents than patients without in-hospital medical complications within 12 months after disease onset.

**Table 3:**
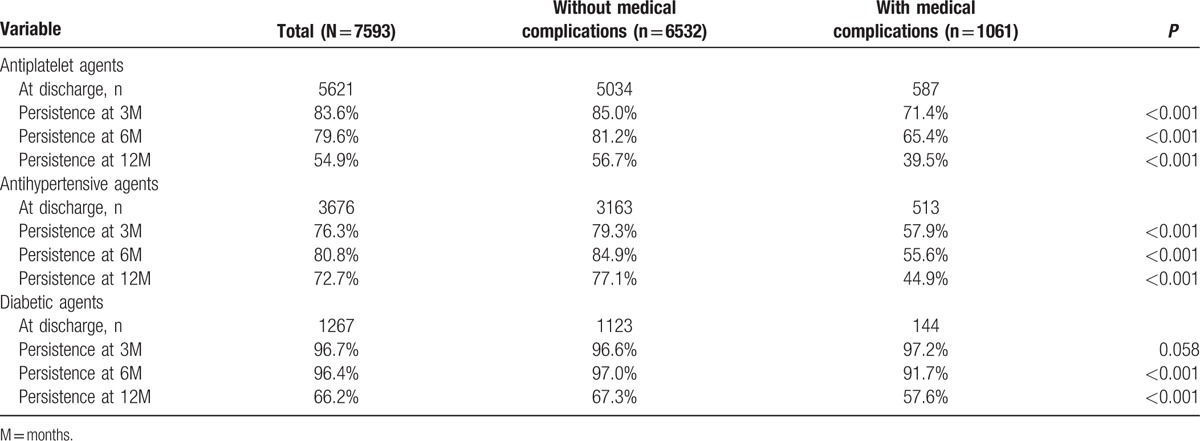
Persistence for secondary prevention medications within 12 months after stroke onset.

## 4 Discussion

The CNSR was a prospective stroke registry that enrolled maximal numbers of stroke patients from the most areas in China so far. In the present study, patients with initial acute ischemic stroke from the CNSR were selected, and the impact of in-hospital medical complications on stroke recurrence was analyzed. In this national prospective cohort, in-hospital medical complications were associated with a higher risk of stroke recurrence in participants with initial ischemic stroke within 12 months after the event. This finding confirms that stroke recurrence in ischemic stroke patients is affected by in-hospital medical complications.

A substantial body of evidence suggests that stroke patients are more susceptible to medical complications and their consequences, such as an increased risk of death or disability.^[24–26,29,36–39]^ Prior studies on the effect of medical complications on stroke outcome mainly focused on death and disability.^[23–31]^ In a recent analysis from the Berlin Stroke Registry study, an effect of acute complications on mortality in ischemic stroke patients was observed, such that the risk of death was 2.5-times higher in participants with in-hospital complications when compared with those without complications.^[27]^ Similarly, we previously showed that the risk of death was significantly higher in stroke patients with in-hospital medical complications than in those without complications, even after adjusting for traditional cerebrovascular risk factors, baseline severity of neurological impairment, anticoagulant treatment, and thrombolytic treatment.^[32]^ Moreover, limited studies aiming at the impact of complications on stroke outcomes demonstrated that patients with complications experienced a higher risk of dependency after stroke than those without complications.^[33,38,40]^ The results of the current study extend these findings by showing that in-hospital medical complications confer an approximately 2-times greater risk of stroke recurrence at 3 months after disease onset, and the risks of recurrent stroke associated with in-hospital medical complications have similar tendencies at 6 months and 12 months after stroke onset. These data suggest that stroke patients with in-hospital medical complications have greater susceptibility to stroke recurrence than those without in-hospital medical complications, which, in turn, may account for much of their excess risk of stroke recurrent events. The reasons why in-hospital medical complications are associated with mortality and disability after stroke onset may be explained by the impact of hospital stays on worsening brain injury, elevated opportunity for recurrent complications, and decreased chance of appropriate post-stroke rehabilitation. The mechanism by which in-hospital medical complications are associated with recurrent stroke is unclear.

Previous studies have found that age,^[7–12]^ race,^[11]^ hypertension,^[8,13–17]^ diabetes mellitus,^[7,9,13,17,18]^ atrial fibrillation,^[8,10,14,16]^ ethanol abuse,^[17,19]^ smoking,^[16]^ hyperlipidemia,^[15]^ elevated homocysteine,^[20]^ patent foramen ovale,^[21]^ and metabolic syndrome^[22]^ predict stroke recurrence. Acupuncture also might play a role in decreasing recurrent stroke events even in ischemic stroke.^[41]^ Medical complications may mediate these predictors of stroke recurrence in patients although the frequency of complications varied widely in the previous studies for various reasons, such as, having the different definitions and types of complications, the varied period for evaluating complications, the different stroke types, and different sample sizes and demographics between studies.^[24–27,32,37,38,40]^ Furthermore, antithrombotic treatments are recommended for secondary prevention after stroke caused by presumed arterial origin, decreasing the risk of recurrent stroke.^[42–47]^ A previous meta-analysis also showed that the risk of recurrent stroke was significantly lower in participants with antihypertensive drugs than those without antihypertensive drugs.^[48]^ Additionally, our previous study confirmed that the risk of stroke recurrence was increased by diabetes.^[18]^ Given that secondary prevention is an important measure to reduce stroke recurrence in stroke patients, a conceivable explanation for our findings is that in-hospital complications may confer a decreased compliance with secondary prevention in participants. Supporting this possibility is the finding that differences in the compliance with secondary prevention were observed between participants with and without in-hospital medical complications. Following stroke onset, participants without complications are more likely to persist in secondary preventive therapies such as aspirin and antihypertensive treatment and antidiabetic treatment, which perhaps partly aggravate differences in the risk for recurrent stroke events. Thus, these factors should be weighed for stroke patients with complications in order to investigate further the mechanisms of stroke recurrence associated with complications and to give proper secondary prevention treatment.

The AVAIL study investigated first the potential factors that were associated with persistence to secondary prevention following stroke.^[49]^ According to the AVAIL study, having insurance to cover medication costs increased by 60% the odds of adherence, which decreased the risk of recurrent stroke. This study also extended the finding that an increased risk of stroke recurrence was associated with no health insurance in patients with initial ischemic stroke before adjusted potential covariates. However, the interesting point of this prospective study was that the risk of stroke recurrence was increased by complications even after adjusted potential covariates, including types of health insurance. In addition, the ratio of having different types of health insurance for patients with complications was similar to that for those without complications in this study. Therefore, this result emphasizes the necessity and importance of specific programs aimed at investigating the mechanisms that underlie complications affecting stroke recurrence in China.

In addition, the different medical complications had different effects on stroke recurrence (Supplementary Table 2). In particular, inflammation may play an important role in stroke recurrence according to our data. Furthermore, we are limited in our current knowledge as to whether effective prevention and treatment of such in-hospital complications can abolish their adverse effects on ischemic stroke recurrence. Therefore, the mechanisms of stroke recurrence associated with in-hospital medical complications need to be further investigated to ascertain these issues.

There are some limitations to this prospective study. This study only included 6 types of complications, and the definitions of some complications were relatively simple. However, all participating hospitals did use the same criteria for diagnosing in-hospital medical complications, and the reported uncertain cases with complications were few. We analyzed data based on a diagnosis of medical complication during hospitalization and did not include information about treatment for medical complications that may be associated with stroke recurrence. In addition, data on newly occurring medical complications were not included during the follow-up period. Thus, we could not address the effects of new medical complications during follow-up on stroke recurrence of patients. This present study did not include whether adequate target levels for blood pressure and glucose had been achieved nor did it collect reasons for medication discontinuation. In addition, information about psychological and cognitive functions after acute stroke was not collected in this study, which may affect behaviors of taking medication(s). Finally, the CNSR study may not represent all levels of hospital expertise because all participating hospitals were from urban regions across China, where investigators may have had more expertise in treating stroke and better medical care resources.

In conclusion, in-hospital medical complications were found to be significantly associated with stroke recurrence after acute ischemic stroke. The decreased adherence to stroke secondary prevention in participants with complications may indirectly cause the elevated events of stroke recurrence. Therefore, future studies aimed at decreasing the rates of stroke recurrence should consider the prevention and treatment of medical complications after stroke. Programs on stroke recurrence need to investigate potential variations carefully, including the impact of adherence to preventive interventions for secondary stroke.

## Supplementary Material

Supplemental Digital Content
