# Supplementary material for: In-hospital medical complications associated with stroke recurrence after initial ischemic stroke: A prospective cohort study from the China National Stroke Registry
Source: Medicine (Baltimore). 2016 Sep 16;95(37):e4929. doi: 10.1097/MD.0000000000004929 (PMC5402614; doi:10.1097/MD.0000000000004929)
Supplement: Supplemental Digital Content [file medi-95-e4929-s001.doc]

**Supplementary material**

Supplementary table 1. Definitions of in-hospital medical complications [^32^](#_ENREF_32) .

| Medical complications | Definition |
| --- | --- |
| Pneumonia | Having fever, and coarse breathing or crackling sounds, confirmed by a chest X-ray |
| Deep vein thrombosis | Having clinical symptoms of deep vein thrombosis, with a deep vein thrombosis by venous Doppler, excluding cellulitis or infection of the leg |
| Pulmonary embolism | Having clinical symptoms of pulmonary embolism, with a deep vein thrombosis in the legs of a patient by venous Doppler, excluding diseases of heart attack, pneumonia, pneumothorax, and dissection of an aortic aneurysm |
| Urinary tract infection | Having clinical symptoms of urinary tract infection with positively presenting abnormal white and red blood cells in urine |
| Gastrointestinal bleeding | Having coffee-ground emesis, hematemesis, and blood in nasogastric tube, melena, or blood in rectum |
| Decubitus ulcer | Having a skin sore (s) resulting from lying in one position too long during hospital |

**Supplementary material**

Supplementary table 2.Unadjusted and adjusted odds ratios of presenting in-hospital medical complications for stroke recurrence following acute ischemic stroke.

| Outcomes | Unadjusted OR (95% CI) | Adjusted OR (95% CI) |
| --- | --- | --- |
| Stroke recurrence at 3M |  |  |
| Pneumonia | 3.00 (2.56-3.51) | 2.18 (1.82-2.63) |
| Urinary tract infection | 2.17 (1.66-2.82) | 1.59 (1.18-2.13) |
| Gastrointestinal bleeding | 3.25 (2.45-4.33) | 2.42 (1.77-3.32) |
| Decubitus ulcer | 4.31 (2.61-7.11) | 2.51 (1.44-4.35) |
| Deep vein thrombosis | 1.20 (0.42-3.43) | 0.83 (0.28-2.47) |
| Pulmonary embolism | 2.16 (0.80-5.84) | 1.40 (0.44-4.39) |
| Stroke recurrence at 6M |  |  |
| Pneumonia | 2.88 (2.48-3.34) | 2.01 (1.69-2.39) |
| Urinary tract infection | 2.41 (1.89-3.07) | 1.65 (1.26-2.16) |
| Gastrointestinal bleeding | 3.21 (2.45-4.20) | 2.26 (1.67-3.06) |
| Decubitus ulcer | 3.77 (2.31-6.17) | 1.99 (1.16-3.43) |
| Deep vein thrombosis | 1.51 (0.62-3.69) | 0.99 (0.39-2.54) |
| Pulmonary embolism | 1.68 (0.62-4.52) | 0.94 (0.30-2.96) |
| Stroke recurrence at 12M |  |  |
| Pneumonia | 2.72 (2.35-3.15) | 1.88 (1.58-2.22) |
| Urinary tract infection | 2.23 (1.76-2.83) | 1.54 (1.18-2.01) |
| Gastrointestinal bleeding | 2.97 (2.27-3.87) | 2.09 (1.55-2.83) |
| Decubitus ulcer | 3.32 (2.03-5.42) | 1.73 (1.00-2.97) |
| Deep vein thrombosis | 1.33 (0.54-3.25) | 0.89 (0.35-2.29) |
| Pulmonary embolism | 1.48 (0.55-3.97) | 0.83 (0.26-2.60) |

Abbreviations: CI (confidence interval), M (months), NIHSS (National Institutes of Health Stroke Scale), OR (odds ratios),TIA (transient ischemic attack).

*Adjusted for age, sex, baseline NIHSS, hypertension, diabetes mellitus, hyperlipidemia, history of coronary heart disease, history of stroke, history of TIA, family history of stroke, atrial fibrillation, current smoking status, dysphagia, heavy alcohol intake, anticoagulant treatment, thrombolytic treatment, and types of health insurance.
